# MicroRNA-873 Suppresses Viability and Invasion of Colorectal Cancer Through KRAS/MAPK Signaling and Sensitizes Tumor Spheroids to 5-Fluorouracil in a 3D Microwell Model

**DOI:** 10.1109/OJEMB.2026.3678868

**Published:** 2026-03-30

**Authors:** Mehrdad Bandegi, Ezgi Biltekin, Yasemin M. Akay, Bulent Ozpolat, Metin Akay

**Affiliations:** ^1^ Department of Biomedical Engineering, Cullen College of EngineeringUniversity of Houston 14743 Houston TX 77204 USA; ^2^ Peggy and Charles Stephenson School of Biomedical Engineering, Gallogly College of EngineeringUniversity of Oklahoma 6187 Norman OK 73019 USA

**Keywords:** Colorectal cancer, KRAS/MAPK, microRNA-873, PEGDA, 3D spheroids, 5-fluorouracil

## Abstract

Colorectal cancer (CRC) ranks third in incidence among all malignancies and is highly lethal in advanced stages. Combination chemotherapy regimens based on 5-fluorouracil (5-FU) remain the mainstay of colorectal cancer treatment alongside surgical resection. Even though new treatment modalities are emerging, many are either ineffective against KRAS-mutant tumors or prone to therapy resistance. Therefore, there is a critical need for new targeted therapies that may overcome the KRAS-driven chemoresistance and enhance the effect of chemotherapy. MicroRNAs can modulate several oncogenic pathways at once and can strengthen chemotherapy. In this study, we identified miR-873 as a potential chemosensitizer that modulates KRAS/MAPK signaling in CRC. We found that KRAS is overexpressed in metastatic versus primary tissues and in a large CRC patient cohort (n 1,061), high KRAS expression was associated with worse overall survival (HR 1.27; 95 CI, 1.041.56; log-rank p 0.018). In vitro inhibition of KRAS by siRNA reduced clonogenic growth (HCT116, p 0.0023; RKO, p 0.0018) and invasion (p 0.0001). In silico prediction (TargetScan/miRWalk) analyses showed a conserved binding site between miR-873 and KRAS 3UTR. Consistent with this prediction, miR-873 mimic transfection reduced KRAS protein expression and phenocopied KRAS knockdown by suppressing colony formation (p 0.0021) and invasion (p 0.0001) in KRAS-mutant HCT116 and KRAS-wild-type RKO cells. Dose-matrix screening and SynergyFinder+ analysis revealed synergistic inhibition of spheroid viability with miR-873 + 5-FU, including a low-dose pair (25 nM miR-873 + 12.5 M 5-FU) showing positive synergy across ZIP/HSA/Bliss/Loewe models. In a poly(ethylene glycol)diacrylate(PEGDA) microwell 3D platform that generates uniform, size-controlled CRC spheroids, this combination produced the strongest suppression of spheroid expansion (day-5/day-3 area: HCT116, 0.61 0.18 vs control, 2.08 0.49; RKO, 0.66 0.04 vs control, 2.08 0.31) and reduced the live-cell fraction to 41 in both lines. Moreover, western blot analysis showed decreased KRAS and MAPK pathway activity (reduced p-ERK and context-dependent p-MEK), reduced Cyclin D1, and increased apoptotic readouts (cleaved PARP and a Bax/Bcl-2 shift). Together, these results position miR-873 treatment as a potential targeting approach to suppress KRAS/MAPK signaling and sensitize CRC to 5-FU and validate our PEGDA microwell 3D platform as a practical, translational testbed for miRNAchemotherapy combinations.

## I. Introduction

Colorectal cancer (CRC) is the third most commonly diagnosed malignancy and the second most prevalent cause of cancer-related mortality worldwide [1]. By 2040, the global colorectal cancer burden is projected to reach approximately 3.2 million new cases and 1.6 million deaths annually [2]. Its public health burden is driven by late presentation, high rates of metastatic spread to liver and lung, and limited durable responses in refractory disease [3], [4]. Five-year survival exceeds 90 for stage I but falls below 15 for stage IV, reflecting the sharp drop in curability once distant metastasis occurs [5].

Current care usually combines surgery with chemotherapeutics based on fluoropyrimidinessuch as 5-fluorouracil (5-FU) or capecitabine, which is often given in combinations such as FOLFOX and FOLFIRI [6], [7]. 5-FU remains a mainstay of CRC chemotherapy. Either singly or in combination as FOLFOX and FOLFIRI, but primary and secondary resistance threaten its long-term sustainability [7], [8]. Even though there are some modalities, including epidermal growth factor receptor (EGFR) or vascular endothelial growth factor (VEGF) therapies, either it is not effective on KRAS mutant tumors or are prone to therapy resistance [7], [8], [9]. Although emerging targeted approaches hold promise as potential new treatment modalities, there is still a critical need for complementary strategies that disable oncogenic signaling while improving chemotherapy sensitivity [8], [9], [10].

Most CRCs arise as adenocarcinomas through the gradual build-up of genomic and epigenetic changes that rewire the RAS/RAF/MEK/ERK (MAPK) signaling pathway [11], [12], [13], [14]. This pathway sustains proliferation, invasion, and therapy resistance [15], [16]. KRAS mutations occur in about 4050 of CRC and are situated at positions G12, G13, and Q61, where aberrant GTPase action keeps KRAS within an active state of conformation and drives persistent RAFMEKERK signaling [17], [18]. MAPK output stimulates G1/S entry through Cyclin D1 (CCND1), and ERK or MEK inhibition reduces Cyclin D1 and suppresses growth [12], [15], [16]. KRAS status also intersects with DNA damage responses and apoptotic control, which regulates the response to combinations targeting repair and survival programs [19], [20], [21]. Clinically, KRAS mutations indicate poorer outcomes, underscoring their role as critical drivers of oncogenic signaling [22], [23].

MicroRNAs (miRNAs) regulate gene expression by sequence-directed silencing of target mRNAs and contribute to tumor initiation, growth, invasion, angiogenesis, and response to therapy in CRC [24], [25], [26], [27], [28]. Their multiplex regulation makes them attractive as biomarkers and as therapeutic agents that can inhibit a plurality of oncogenic pathways at once [25], [26]. miRNA-based therapy can modulate nucleotide stress signaling and survival mechanisms that govern 5-FU sensitivity [28], [29], [30].

Two-dimensional culture is informative but cannot capture the gradients, extracellular matrix restriction, and architecture that direct drug response in vivo. Increased predictive pharmacologic behavior can be achieved with three-dimensional spheroid and hydrogel platforms [31], [32], and patient-derived organoids forecast chemotherapy response in metastatic CRC [34]. Prior 3D studies have established heterotypic CRC spheroids as robust drug-testing models [35], and our poly(ethylene glycol) diacrylate (PEGDA) microwell platform has demonstrated microRNA-driven chemosensitization, supporting microwell spheroids as practical testbeds for microRNAchemotherapy combinations [43].

In this study, we targeted colorectal cancer cells with miR-873 treatment alone or in combination with the chemotherapeutic agent 5-FU. We investigated the tumors suppressive effect on 2D cell viability, colony formation, and metastasis. Furthermore, we examined the synergistic tumor suppressive effect of miR-873 and 5-FU combination on CRC tumor spheroids in our 3D PEGDA-based platform through inhibition of the KRAS signaling. Our analysis of a CRC patient dataset (n1061) showed that high KRAS expression is associated with poor prognosis. Functionally, KRAS inhibition by siRNA suppresses the tumors viability, colony formation, and invasion in CRC cells. TargetScan analysis indicated that miR-873 is predicted to bind a conserved site in the KRAS 3UTR. Furthermore, when we administered miR-873, it reduced the viability, colony formation, and invasion ability of KRAS-mutant HCT116 and KRAS-wild-type RKO CRC cells. In 3D, CRC spheroids treated with miR-873, alone or combined with 5-FU, responded synergistically and cell viability decreased at low combinational doses such as 25 nM miR-873 with 12.5 M 5-FU. Using our 3D PEGDA microwell platform, we tracked spheroid size and found that the optimal miR-873 + 5-FU combination markedly suppressed spheroid growth, downregulated KRAS signaling, and increased apoptosis. Overall, miR-873 treatment suppresses colony formation and invasion in 2D models and sensitizes 3D spheroids to low-dose 5-FU by reducing KRASMAPK signaling, decreasing Cyclin D1 expression, and inducing apoptosis.

## II. Materials and Methods

### A. Cell Lines and Culture

HCT116 (KRAS^G13D) and RKO (KRAS^WT) colorectal cancer cell lines were obtained from the American Type Culture Collection (ATCC, Manassas, VA, USA). HCT116 cells were maintained in McCoys 5A medium (ATCC) supplemented with 10 fetal bovine serum (FBS) and 1 penicillinstreptomycin (10000 U/mL; Thermo Fisher Scientific, Waltham, MA, USA). RKO cells were maintained in Eagles Minimum Essential Medium (EMEM; ATCC) with 10 FBS and 1 penicillinstreptomycin. Cultures were kept in a humidified incubator at 37 C with 5 CO_2_.

### B. miRNA/siRNA Transfection

miR-873 mimic and control miRNA (Thermo Fisher Scientific) and KRAS siRNA with non-targeting control (Sigma-Aldrich, St. Louis, MO, USA) were delivered using HiPerFect (Qiagen, Germantown, MD, USA). Cells were seeded (12-well: 300 cells/well for clonogenic assays; 6-well: 5.0 10^4^4.0 10^5^ cells/well for protein/functional assays), then transfected per manufacturers protocol. Unless noted, 25 nM miR-873 or siRNA was used (screens: miRNA 0100 nM; siRNA 0100 nM). Cells were incubated 5 days post-transfection before downstream assays.

### C. Protein Extraction and Immunoblotting

Cells were harvested after 10 days of total culture (5 days of transfection + 5 days 3D/treatment). Pellets were lysed in ice-cold RIPA with protease/phosphatase inhibitors (Thermo Fisher Scientific); protein was quantified by Pierce BCA protein Assay kit (Thermo Fisher Scientific). Equal protein (60 g) was resolved on 12 Mini-PROTEAN TGX gels (Bio-Rad, Hercules, CA, USA) and transferred to PVDF (Thermo Fisher Scientific). Membranes were blocked (5 milk in TBS-T; Bio-Rad) and probed overnight with primary antibodies: KRAS (Santa Cruz Biotechnology, Dallas, TX, USA); MEK1/2, p-MEK1/2 (Ser217/221), ERK1/2, p-ERK1/2 (Thr202/Tyr204), Cyclin D1, PARP, cleaved-PARP, Bax, Bcl-2, GAPDH/-actin (Cell Signaling Technology, Danvers, MA, USA) in 5 milk or 5 Bovine Serum Albumin (BSA; Thermo Fisher Scientific). HRP-conjugated secondaries (Cell Signaling Technology) were applied for 1 h; blots were developed with SignalFire Plus ECL (Cell Signaling Technology) and imaged on ChemiDoc (Bio-Rad) with matched exposures. When needed, membranes were stripped (Restore PLUS, Thermo Fisher Scientific) and reprobed. Densitometry was performed in Image Lab (Bio-Rad); bands were normalized to GAPDH or -actin.

### D. Colony Formation Assay

Cells (300/well, 12-well) were transfected (25 nM miR-873 or negative control; or 25 nM KRAS siRNA or non-targeting siRNA) with HiPerFect (Qiagen) and grown 710 days. Colonies were fixed/stained with crystal violet (Thermo Fisher Scientific), imaged, and counted in ImageJ (global threshold; Analyze Particles). Counts were normalized to matched controls; data are mean SD from biological replicates.

### E. Matrigel Invasion (Transwell) Assay

After 5 days of transfection (25 nM miR-873 or control; 25 nM KRAS siRNA or control), 8 10^4^ cells in serum-free medium were seeded into Matrigel-coated Transwell inserts (24-well, 8.0 m pores; Falcon/Corning, NY, USA). The lower chambers contained medium supplemented with 10 FBS as a chemoattractant. Cultures were maintained at 37 C, 5 CO_2_, and at the endpoint, non-invaded cells were removed from the upper surface; membranes were fixed and stained with the Hema 3 system (Fisher Healthcare/Fisher Scientific), mounted, and imaged. Invaded cells on the lower surface were counted in five non-overlapping fields per insert by using a Zeiss Axiovert 200 M inverted fluorescence microscope (Carl Zeiss, Oberkochen, Germany), and values were normalized to matched controls; data are presented as mean SD. This assay followed our previously reported Matrigel-coated Transwell protocol (with the same fix/stain and field-counting procedures) [37].

### F. Drug Preparation and Treatments

5-Fluorouracil (5-FU; Sigma-Aldrich, St. Louis, MO, USA) was prepared as a 100 mM stock in dimethyl sulfoxide (DMSO; Santa Cruz Biotechnology, Dallas, TX, USA) and diluted freshly into complete growth medium before use. For matrix screening, 5-FU was tested at 0, 6.25, 12.5, 25, 50, and 100 M. For all follow-up assays, cells were treated with 12.5 M 5-FU for 48 h, either as a single agent or following miR-873 transfection.

### G. Viability Matrix and Synergy Analysis

After 5 days of transfection (0, 12.5, 25, 50, 100 nM miR-873), cells were detached with Trypsin-EDTA 0.05 (ATCC), counted (NanoEntek slides; Invitrogen Countess, Thermo Fisher Scientific), and seeded (1500 cells/well) in ultra-low-attachment U-bottom 96-well Nunclon Sphera plates (Thermo Fisher Scientific). Spheroids formed for 3 days (baseline images, day 3), then were treated with 5-FU (0100 M) for 48 h (images day 5). Viability was measured using PrestoBlue HS (Thermo Fisher Scientific), incubated for 3 h at 37 C, and fluorescence was read on a BioTek Synergy H1 multimode plate reader (Agilent BioTek, Winooski, VT, USA); signals were background-subtracted and expressed as inhibition 100 viability normalized to control miRNA. Drug interaction was quantified in SynergyFinder+ (ZIP, HSA, Bliss, Loewe) on mean inhibition matrices from 3 biological replicates.

### H. 3D Spheroid Generation in PEGDA Microwell Chips

Square glass coverslips (24 24 mm^2^) were silanized with 3-(trimethoxysilyl)propyl methacrylate (TMSPMA; Sigma-Aldrich, St. Louis, MO, USA). Slides were surface activated in sodium hydroxide solution (NaOH; Thermo Fisher Scientific, Waltham, MA, USA) overnight, rinsed several times with ethanol and deionized water, and baked for 1 h to dry. TMSPMA (about 3 mL total for 40 slides) was applied to the surfaces. Slides were wrapped in aluminum foil and baked overnight. Excess silane was removed with multiple ethanol rinses followed by a 1 h bake. Treated slides were stored dry until use. A photopolymerizable solution was prepared at 40 (w/v) PEGDA (MW 700; Sigma-Aldrich) in phosphate-buffered saline (PBS; Sigma-Aldrich) with 0.2 (w/v) 2-hydroxy-4-(2-hydroxyethoxy)-2-methylpropiophenone as the photoinitiator. The mixture was protected from light and placed on a room-temperature shaker overnight to dissolve completely. On TMSPMA-treated glass, a base coat of PEGDA (20 L) was dispensed and polymerized under a UV spot source (OmniCure Series 2000, Lumen Dynamics Group Inc., Mississauga, ON, Canada) for 35 s. A second layer of PEGDA (200 L) was added, covered with a chrome photomask (CAD/Art Services, Bandon, OR, USA) patterned with a square array of 600 m diameter circular features, and exposed for 36 s to form a microwell array. Chips were transferred to 6-well plates, immersed in sterile PBS, rinsed once, and equilibrated overnight at 37 C with 5 CO_2_. The next day, chips were rinsed again with PBS immediately before seeding. HCT116 and RKO monolayers that had been transfected in 2D were detached, counted, and concentrated. For each chip, residual PBS was removed. A 170 L cell suspension containing approximately 0.4 10^6^ cells was gently dispensed to cover the chip. Cells were allowed to settle into the wells for 5 min at room temperature. Complete growth medium (1830 L) was then added along the side of the well to a total of 2 mL, ensuring the chips remained submerged without disturbing settled cells. Chips were incubated at 37 C in 5 CO_2_. Spheroids formed undisturbed for 3 days. Bright-field images were acquired on day 3 to establish the pre-treatment baseline. 5-FU doses were then added at the indicated dose, and cultures were returned to the incubator for 48 h. On day 5, post-treatment images were captured, and spheroids were collected for downstream analyses.

### I. Cell Viability and Spheroid Size (3D chips)

Conditioned medium was collected; chips were rinsed with PBS; spheroids were dissociated with TrypsinEDTA 0.05 (Thermo Fisher Scientific) (4 min, 37 C) and quenched with complete medium. After centrifugation (1500 rpm, 5 min), pellets were resuspended (1.0 mL). Trypan Blue 0.4 (Thermo Fisher Scientific) counts were obtained on the Invitrogen Countess (Thermo Fisher Scientific) for live, dead, and total cells (n3 chips/condition). Bright-field images (Olympus, Tokyo, Japan) were captured on day 3 and day 5 under identical settings. Spheroid area was quantified in ImageJ/Fiji; day-5/day-3 area ratios were normalized to control miRNA. Data are mean SD from 3 independent chips.

### J. Statistical Analysis

Data are presented as mean SD. Unless otherwise stated, results are from three independent biological experiments, each performed in technical triplicate. Two-group comparisons used unpaired, two-tailed Students t-tests; multi-group (3) comparisons used one-way ANOVA with appropriate post hoc tests for multiple comparisons. Drug-combination matrices were analyzed in SynergyFinder+ to report ZIP, HSA, Bliss, and Loewe synergy scores and CSS/RI metrics. KaplanMeier curves were compared by log-rank test, and TNMplot tumornormalmetastatic differences by KruskalWallis with Dunns post hoc tests when applicable. p < 0.05 was considered statistically significant. Analyses were performed in GraphPad Prism v9.5.1.

## III. Results

### A. KRAS Expression and MAPK Pathway Activation Are Associated With Poor Prognosis in Colorectal Cancer

**FIGURE 1.**
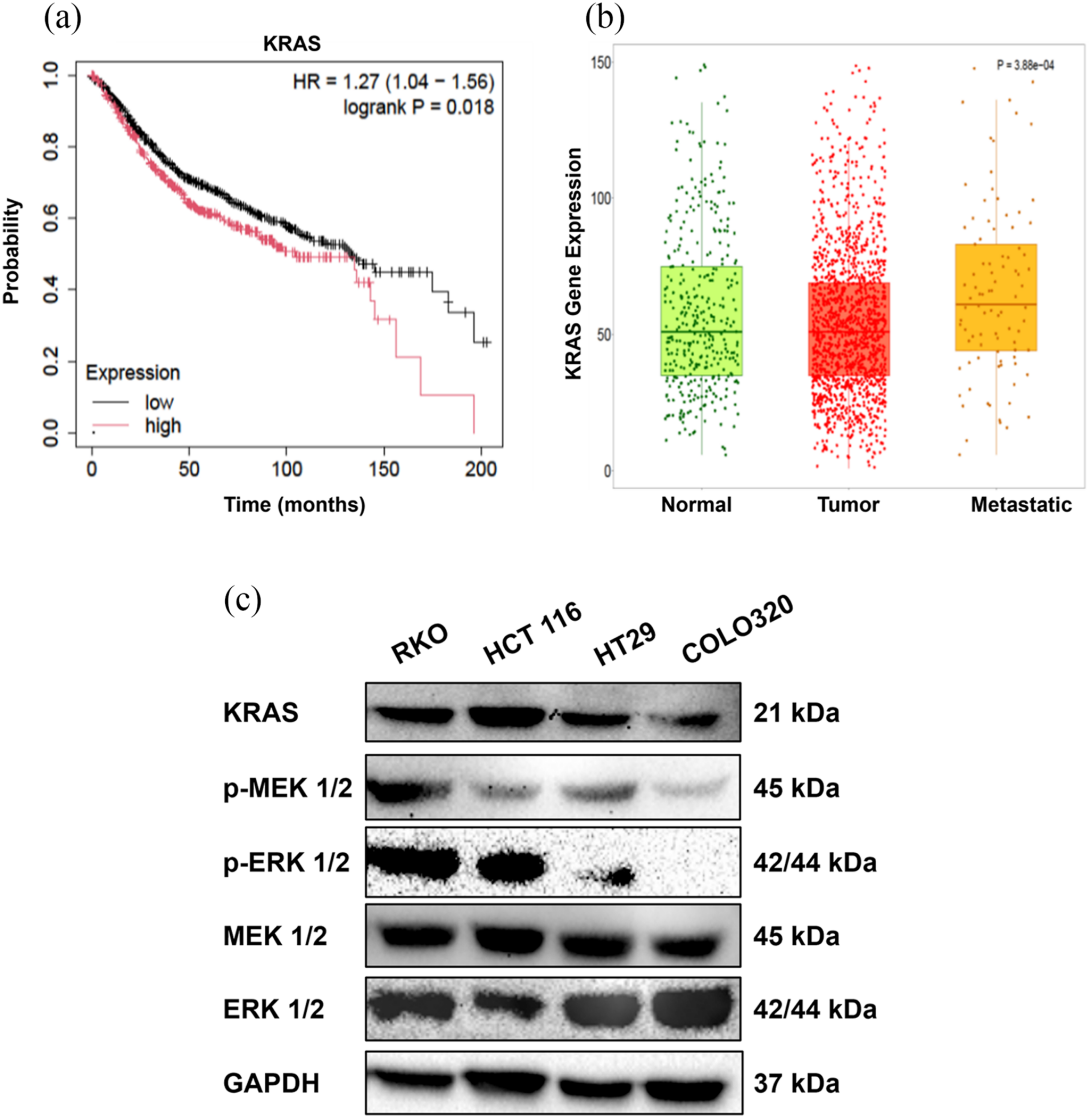
KRAS level associates with poor outcome and MAPK activation in CRC. (a) KaplanMeier overall-survival curves for CRC patients stratified by KRAS expression (high vs. low); hazard ratio (HR) 1.27 (95 CI, 1.041.56); log-rank *p* 0.018. (b) TNMplot gene-chip data showing KRAS mRNA across normal, primary tumor, and metastatic colon tissues (KruskalWallis *p* 3.88 10^4^); metastatic tissues display a further increase versus tumor. (c) Immunoblot of KRAS and MAPK proteins (p-MEK1/2, p-ERK1/2, total MEK1/2, total ERK1/2) in CRC cell lines (RKO, HCT116, HT29, COLO320HSR); KRAS, p-MEK, and p-ERK are higher in RKO/HCT116; GAPDH, loading control.

Publicly available CRC patient datasets were queried to examine the clinical significance of KRAS [38]. KaplanMeier survival analysis showed that patients with high KRAS expression had significantly worse overall survival than those with low expression (HR 1.27; 95 CI, 1.041.56; log-rank p 0.018; Fig. 1(a)), with median survival of 105 months in the high-expression group versus 134 months in the low-expression group. Expression profiling with the TumorNormalMetastatic plot (TNMplot, gene-chip data) showed that KRAS messenger RNA (mRNA) differs across normal, primary tumor, and metastatic colon tissues (KruskalWallis p 3.88 10^4^) [39]. Metastatic tissues showed a further elevation in expression (Fig. 1(b)). Post hoc tests confirmed differences in normaltumor (p 1.58 10^2^), normalmetastatic (p 1.50 10^2^), and tumormetastatic (p 1.89 10^4^) comparisons. These observations indicate a gradual induction of KRAS transcription during disease progression. Western blot results showed KRAS protein was expressed at various levels in all four CRC cell lines (RKO, HCT116, HT29, and COLO320), with the highest expression in RKO and HCT116 (Fig. 1(c)). p-MEK and p-ERK were higher in RKO and HCT116, but total MEK and ERK were equal in all samples. GAPDH was used as the loading control.

Together, the protein and transcript analyses showed that KRAS expression is elevated in metastatic CRC patients tissues in comparison with normal tissue, and its overexpression is associated with more robust MEK/ERK pathway activity. Patients with high levels of KRAS have poorer survival, indicating its importance in CRC progression.

### B. KRAS Knockdown and miR-873 Restoration Inhibit Cell Growth and Invasion in Colorectal Cancer Cells

**FIGURE 2.**
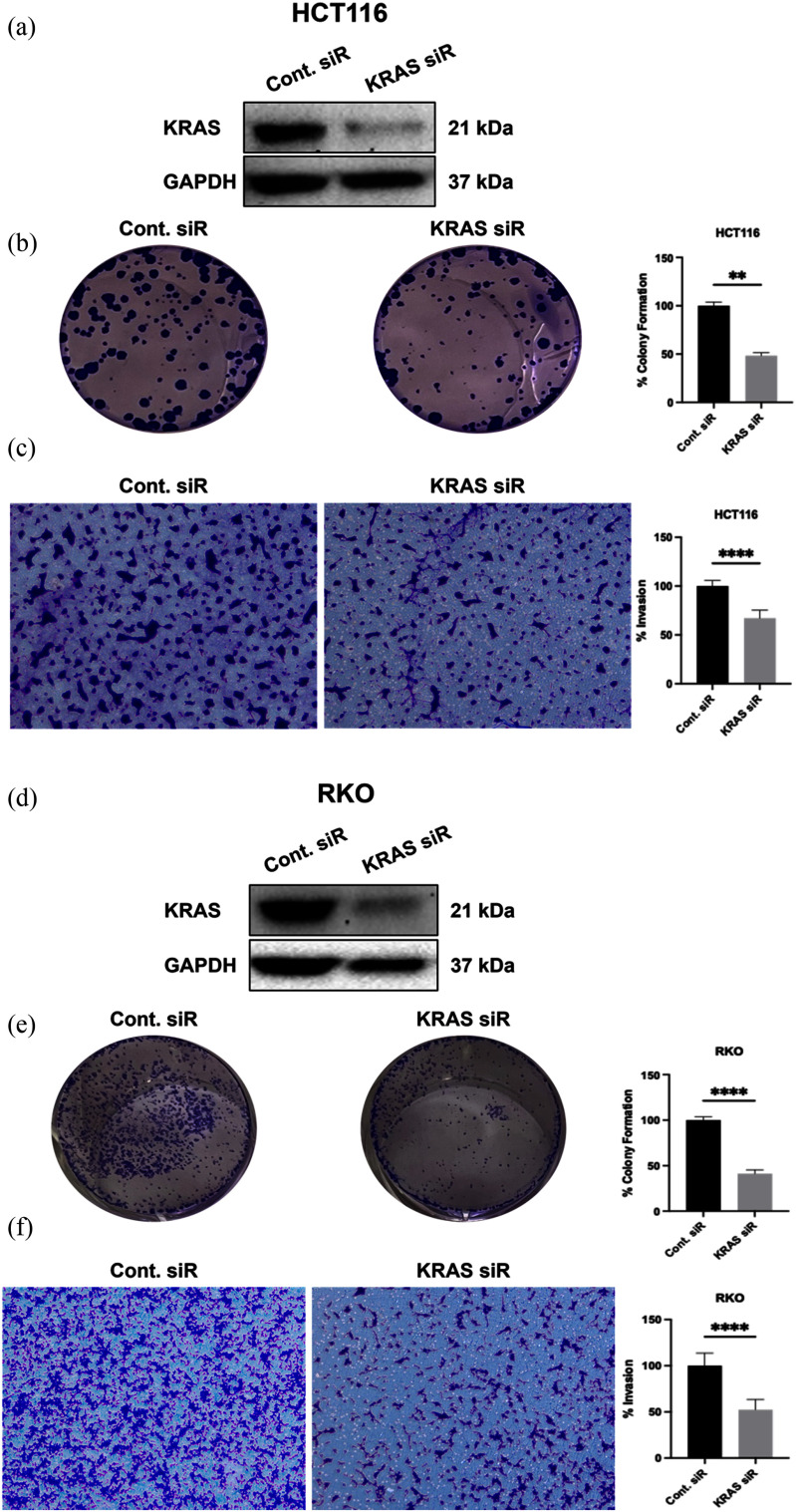
KRAS knockdown suppresses clonogenic growth and invasion. HCT116 (left) and RKO (right) were transfected with KRAS siRNA or control siRNA. (a,d) Western blots confirming KRAS depletion (GAPDH loading control). (b,e) Representative colony images and quantification of colony area; KRAS siRNA significantly reduces clonogenicity (HCT116, p 0.0023; RKO, p 0.0018). (c,f) Transwell-Matrigel invasion images and quantification; KRAS knockdown markedly decreases invasion in both cell lines (*p* 0.0001).

We selected HCT116 (KRAS-mutant) and RKO (KRAS-wild-type) CRC cells to evaluate the effect of KRAS on colorectal cancer growth. RKO and HCT116 cells were transfected with siRNA targeting KRAS. Western blot analysis showed a dramatic decrease in KRAS protein levels in comparison with controls (Fig. 2(a), (d)). Reduced KRAS expression inhibited colony formation in both cell lines (Fig. 2(b), (e)). Analysis of colony areas showed that colonies declined significantly in treatment groups in comparison to the control in HCT116 (p0.0023) and in RKO (p0.0018). To better understand the effect of KRAS on CRC metastasis ability, we conducted a Transwell Matrigel assay. HCT116 and RKO cells were transfected with KRAS siRNA and seeded into Matrigel-coated Transwells. We observed that inhibition of KRAS significantly suppressed the invasion ability of HCT116 and RKO cells (p 0.0001) (Fig. 2(c), (f)). Loss of both growth and invasion indicates that KRAS is an important player for the proliferation and motility of colorectal cancer cells.

### C. miR-873 Suppresses KRAS expression, Cell Growth, and Invasion

**FIGURE 3.**
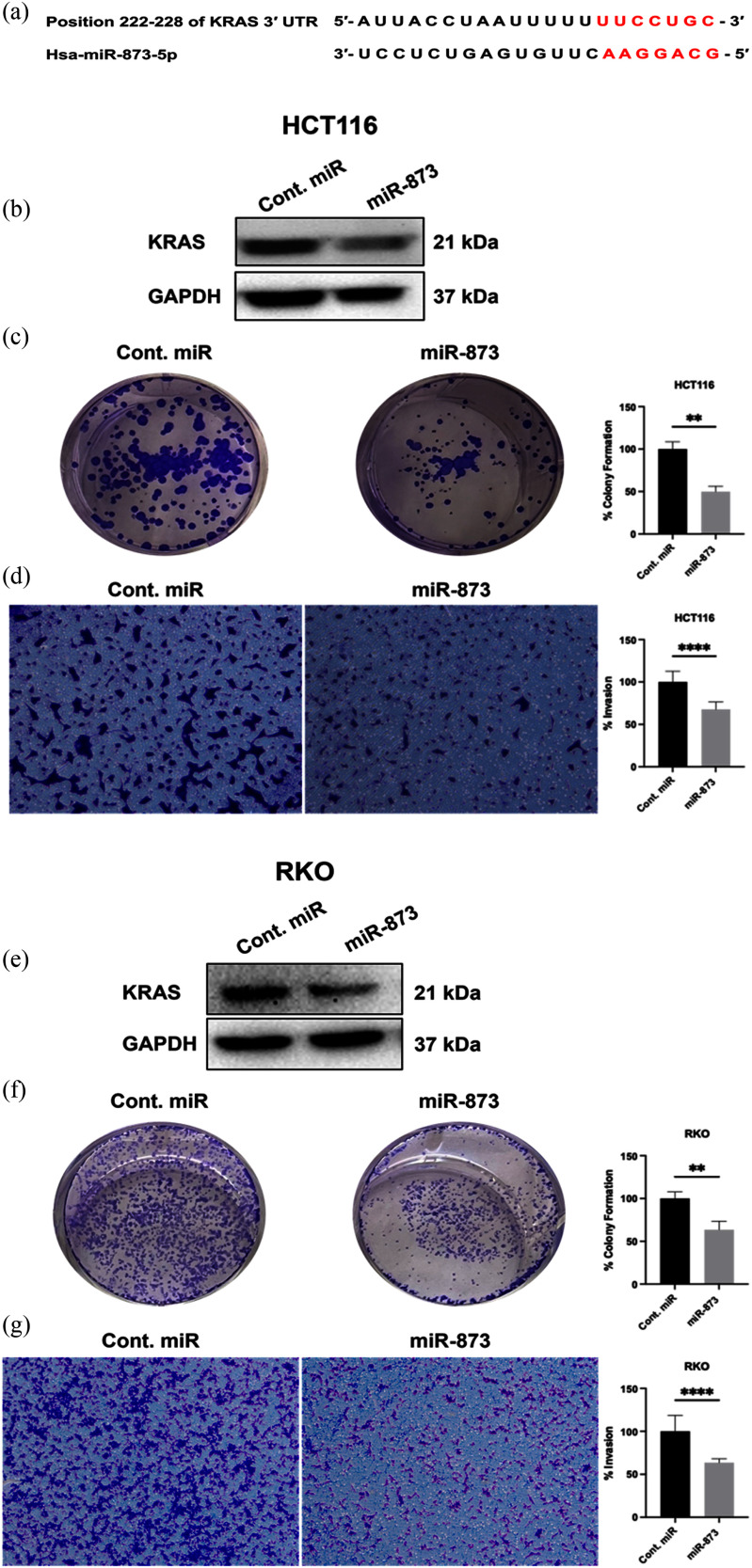
miR-873 suppressed KRAS expression and inhibited CRC growth and invasion (a) Schematic of the conserved miR-873 binding site in the human KRAS 3UTR (seed match indicated) and mutant control. (b,e) Immunoblots showing reduced KRAS protein after miR-873 mimic transfection in HCT116 and RKO (GAPDH control). (c,f) Colony formation is significantly decreased by miR-873 (HCT116, p 0.0015; RKO, p 0.0021). (d,g) Transwell-Matrigel invasion is strongly suppressed by miR-873 in both lines (*p* 0.0001).

To explore potential KRAS regulation by miR-873, we performed in silico target prediction analysis using TargetScan and miRWalk [40]. These analyses identified a conserved miR-873 binding site within the human KRAS mRNA 3 untranslated region (Fig. 3(a)). Consistent with this prediction, transfection with a miR-873 mimic reduced KRAS protein expression in both cell lines (Fig. 3(b), (e)). miR-873 significantly suppressed colony formation in HCT116 (p 0.0015) and RKO (p 0.0021) (Fig. 3(c), (f)) and reduced invasion in Matrigel-coated Transwells (p 0.0001) (Fig. 3(d), (g)). The pattern of functional suppression was similar to that observed after KRAS siRNA treatment, suggesting that miR-873-mediated effects are consistent with downregulation of KRAS signaling. Collectively, these findings support a regulatory relationship between miR-873 and KRAS that is consistent with predicted KRAS 3UTR targeting and downstream inhibition of CRC proliferative and invasive phenotypes.

### D. miR-873 Treatment Synergistically Enhances the Efficacy of 5-FU in Colorectal Cancer Cells

**FIGURE 4.**
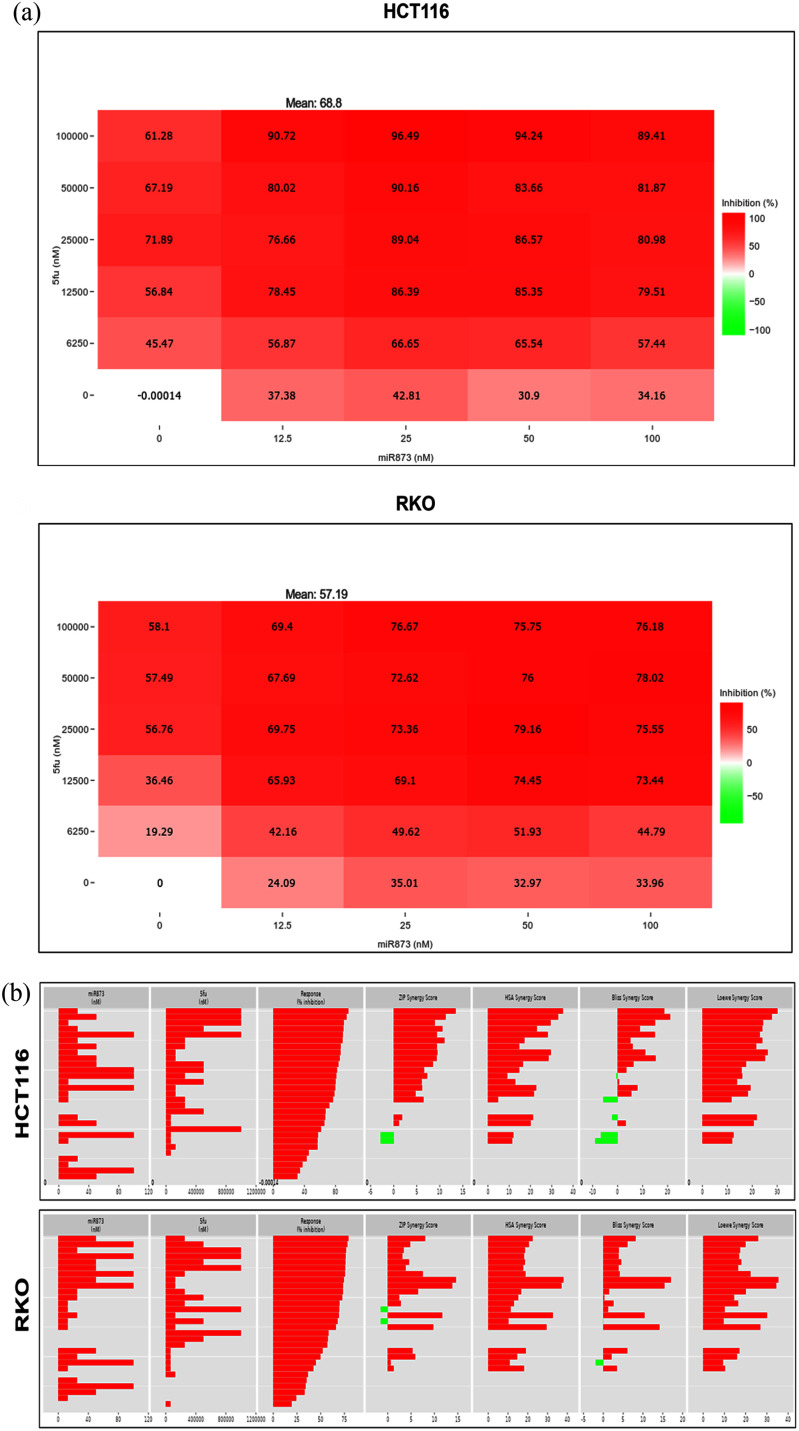
Matrix screens identify synergistic miR-873 + 5-FU dose regions. (a) SynergyFinder+ inhibition heatmaps for miR-873 (12.5100 nM) 5-FU (6.25100 M) in HCT116 (left) and RKO (right); higher inhibition shown in deeper red. Mean inhibition across all combinations: HCT116, 68.8; RKO, 57.2. (b) Waterfall plots summarizing model-specific synergy per combination (ZIP, HSA, Bliss, Loewe) for HCT116 and RKO, highlighting additive/synergistic windows across the matrices.

5-FU remains a mainstay of CRC chemotherapy, used alone or in combinations such as FOLFOX and FOLFIRI; however, primary and acquired resistance limit the durability of response and motivate sensitization strategies. MicroRNA-based approaches can modulate nucleotide-stress signaling and survival programs that influence 5-FU sensitivity. To test whether miR-873 enhances 5-FU response, HCT116 and RKO cells were first transfected with a miR-873 mimic with different doses (12.5 nM, 25 nM, 50 nM, and 100 nM), then seeded into ultra-low-attachment U-bottom 96-well plates to form single, uniform spheroids. After 72 h of spheroid development, cultures were treated with graded 5-FU doses (at 0, 6.25, 12.5, 25, 50, and 100 M), and outcomes were assessed 48 h later. Spheroid area change was quantified from bright-field images, and metabolic viability was measured by using a resazurin-based fluorescent viability assay (PrestoBlue HS reagent). The two-drug test was performed with SynergyFinder+ [41], [42], [43], [44]. The matrix included miR-873 and 5-FU doses. HCT116 and RKO cells were treated in every combination, and cell viability was measured to calculate inhibition values. Both cell lines showed clear dose-dependent decreases in viability. The mean inhibition across all treatment pairs was 68.8 for HCT116 and 57.2 for RKO. The stronger inhibition in HCT116 reflected the increased sensitivity of the KRAS-mutant line to the combination therapy (Fig. 4(a)). Drug interaction strength was quantified using the ZIP, HSA, Bliss, and Loewe models in SynergyFinder+ (Fig. 4(b)).

**FIGURE 5.**
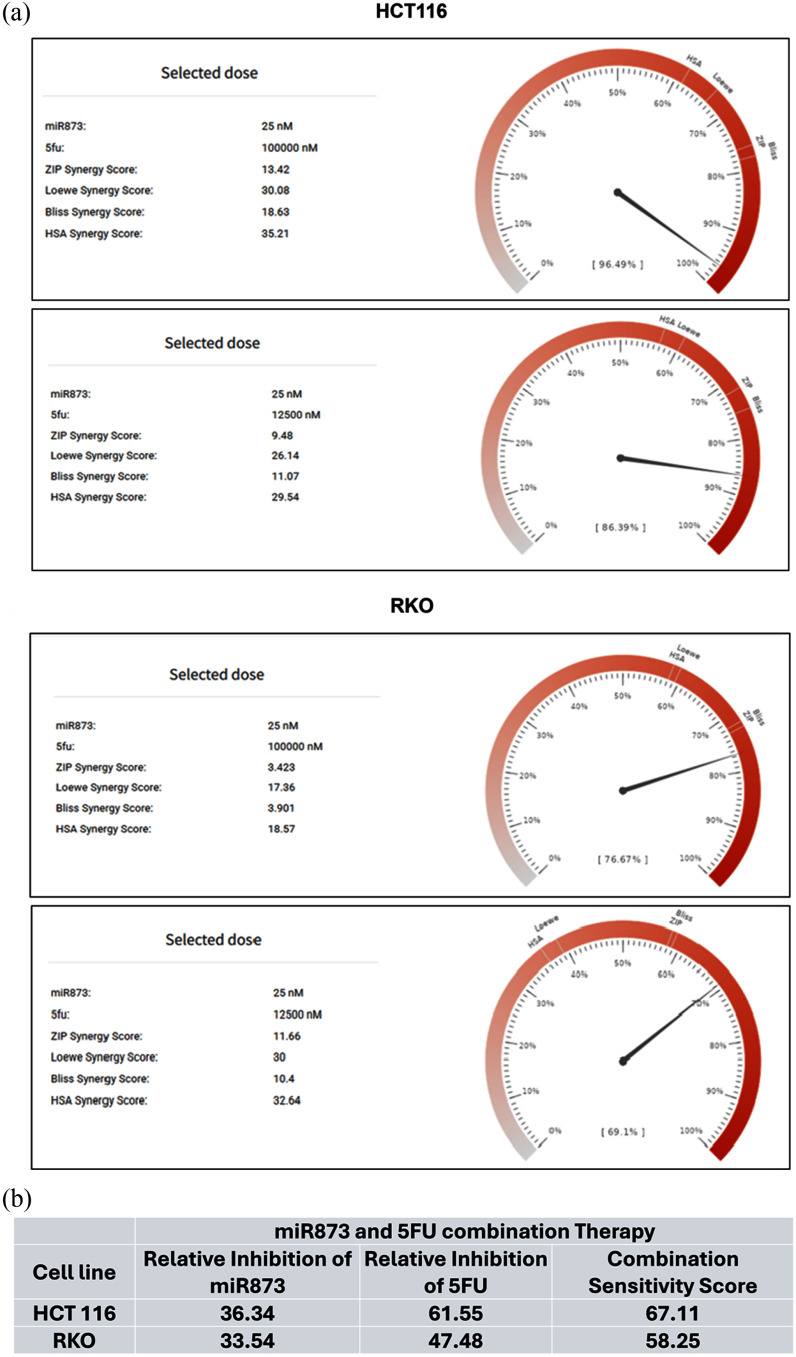
Dose selection and global sensitivity metrics for the miR-873/5-FU combination. (a) Selected dose gauges from SynergyFinder+ for the most effective pairs per cell line, reporting ZIP, Loewe, Bliss, and HSA synergy scores at those doses. (b) Relative inhibition (RI) for the single agents and combination sensitivity score (CSS) for the pair in each line. HCT116: RI (miR-873) 36.34, RI (5-FU) 61.55, CSS 67.11. RKO: RI (miR-873) 33.54, RI (5-FU) 47.48, CSS 58.25. CSS exceeds single-agent RI, supporting synergy.

Positive synergy scores were generated by all models, which suggested that the combination had cooperative effects. The ZIP model assumes interaction without mutual influence. The HSA model compares the observed effect with that of the most effective single agent. The Bliss and Loewe models evaluate independent and equal dose responses, respectively. In HCT116, the combination of 25 nM miR-873 with 100 M 5-FU gave synergy values of 13.42 (ZIP), 35.21 (HSA), 18.63 (Bliss), and 30.08 (Loewe). The combination of 12.5 M 5-FU and 25 nM miR-873 gave 9.48, 29.54, 11.07, and 26.17. In RKO, synergy was modest but reproducible. The higher dose combination gave 3.42, 18.57, 3.90, and 17.36, and the lower dose combination 11.66, 32.64, 10.40, and 30.05 in the same models (ZIP, HSA, Bliss, and Loewe scores respectively) (Fig. 5(a)). Relative inhibition (RI) and combination sensitivity score (CSS) were also determined (Fig. 5(b)). For HCT116, miR-873 alone reduced viability by 36.34 , 5-FU alone by 61.55 , and the combination had a CSS of 67.11. In RKO, RI values were 33.54 for miR-873 and 47.48 for 5-FU, with a CSS of 58.25. In both lines, CSS was higher than single-agent RI values, suggesting synergy between miR-873 and 5-FU. Based on these results, 25 nM miR-873 and 12.5 M 5-FU were selected for utilization in subsequent experiments. This concentration combination produced reproducible synergy and consistent inhibition in both cell models. The results indicate that miR-873 synergistically enhances the suppressive activity of 5-FU on cell viability, with HCT116 cells having a greater response.

### E. Synergistic Effect of miR-873 in Response to 5-FU on Spheroid Growth and Viability in Colorectal Cancer Cells Reduction

**FIGURE 6.**
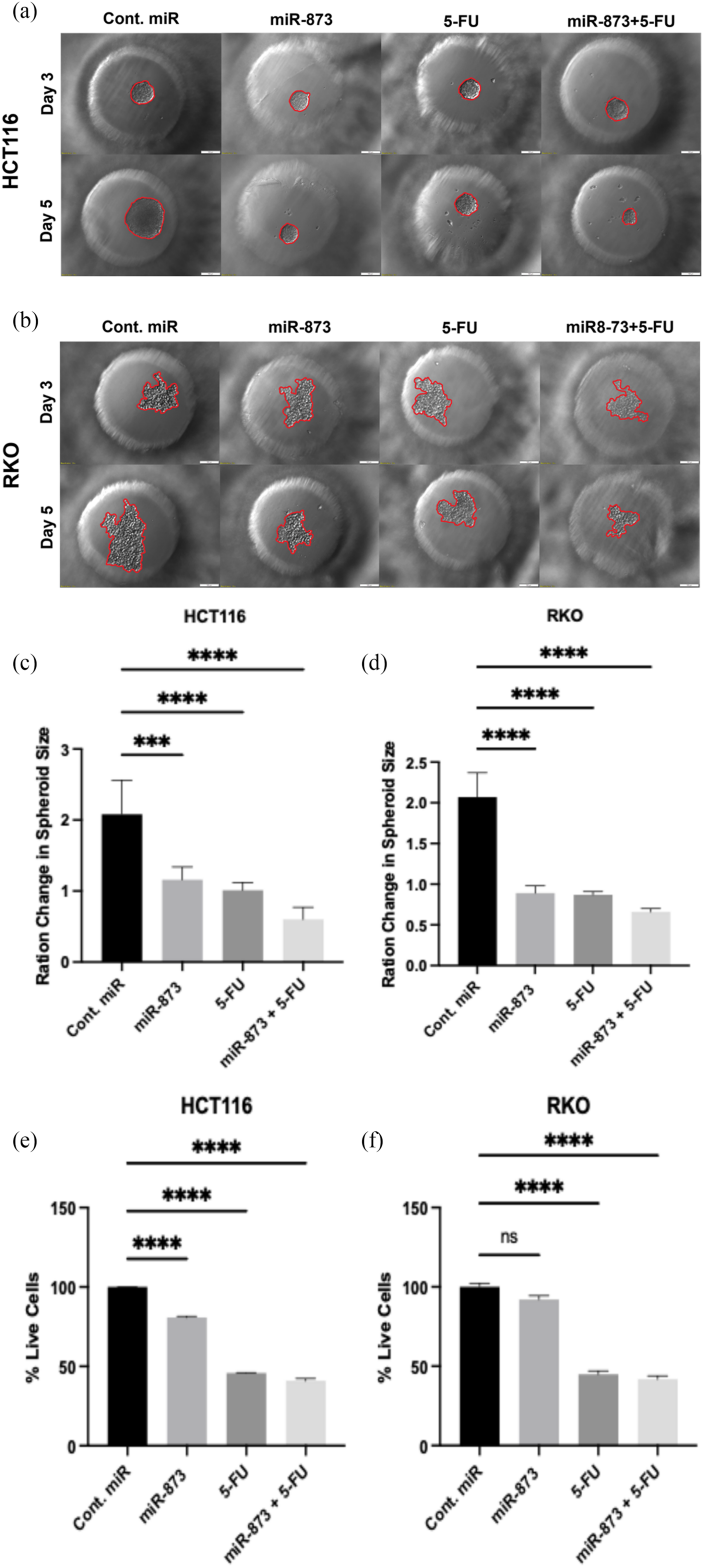
miR-873 + 5-FU reduces spheroid expansion and viability in PEGDA microwells. (a,b) Bright-field images of HCT116 (a) and RKO (b) spheroids at day 3 (pre-treatment) and day 5 after 5-FU (12.5 M) with or without prior miR-873 (25 nM); red outlines indicate spheroid boundaries. (c,d) Fold-change in spheroid area (day-5/day-3), normalized to control miR: HCT116control 2.08 0.49, miR-873 1.16 0.18, 5-FU 1.01 0.12, combo 0.61 0.18; RKOcontrol 2.08 0.31, miR-873 0.90 0.10, 5-FU 0.87 0.05, combo 0.66 0.04. (e,f) Percentage of live cells by Trypan Blue (normalized to control): HCT116miR-873 80.79 0.77, 5-FU 45.77 0.34, combo 40.89 1.31; RKOmiR-873 92.10 2.28, 5-FU 44.98 2.12, combo 41.85 2.09 (ns, not significant; p < 0.001; p < 0.0001).

After determining the highest synergistic combination score of miR-873 and 5-FU treatment, to understand its effect on spheroid growth and viability, HCT116 and RKO cells were first transfected with miR-873 at 25 nM for five days under 2D culture conditions. Transfected cells were next seeded into PEGDA microwell chips to form uniform 3D spheroids. After 3 days of spheroid development, bright-field images were taken (day 3), and then 5-FU treatment (12.5 M) was performed. After two days of treatment, the spheroids were imaged again on day 5, and the area change was measured by ImageJ (Fig. 6(a), (b)).

In HCT116 spheroids, the control transfection (Cont. miR) increased spheroid size to 2.08 0.49-fold, whereas miR-873 alone limited growth to 1.16 0.18, 5-FU alone to 1.01 0.12, and the miR-873 + 5-FU combination produced the strongest inhibition at 0.61 0.18 (Fig. 6(c)). Phenotypically, control spheroids were rounded and compact, while treated spheroidsespecially under the combinationwere smaller, less cohesive, and irregular. A similar pattern was observed in RKO spheroids: Cont. miR 2.08 0.31-fold, miR-873 0.90 0.10, 5-FU 0.87 0.05, and miR-873 + 5-FU 0.66 0.04 (Fig. 6(d)). As in HCT116, treatments yielded distorted spheroid morphology with disrupted cellcell organization, consistent with compromised structural integrity. Cell viability was quantified by Trypan Blue exclusion on the Invitrogen Countess (Fig. 6(e), (f)), and values were normalized to the control miR (100). In HCT116, miR-873 alone reduced the live fraction to 80.79 0.77, 5-FU to 45.77 0.34, and the combination to 40.89 1.31. In RKO, miR-873 yielded 92.10 2.28, 5-FU 44.98 2.12, and the combination 41.85 2.09. Across both lines, the dual treatment produced a further decrease versus 5-FU alone, with the largest effect in KRAS-mutant HCT116, consistent with miR-873 restoration enhancing 5-FU chemosensitivity in colorectal cancer cells. (p < 0.05)

### F. miR-873 Suppresses KRAS/MAPK Signaling and Enhances 5-FU Sensitivity in Colorectal Cancer Cells

**FIGURE 7.**
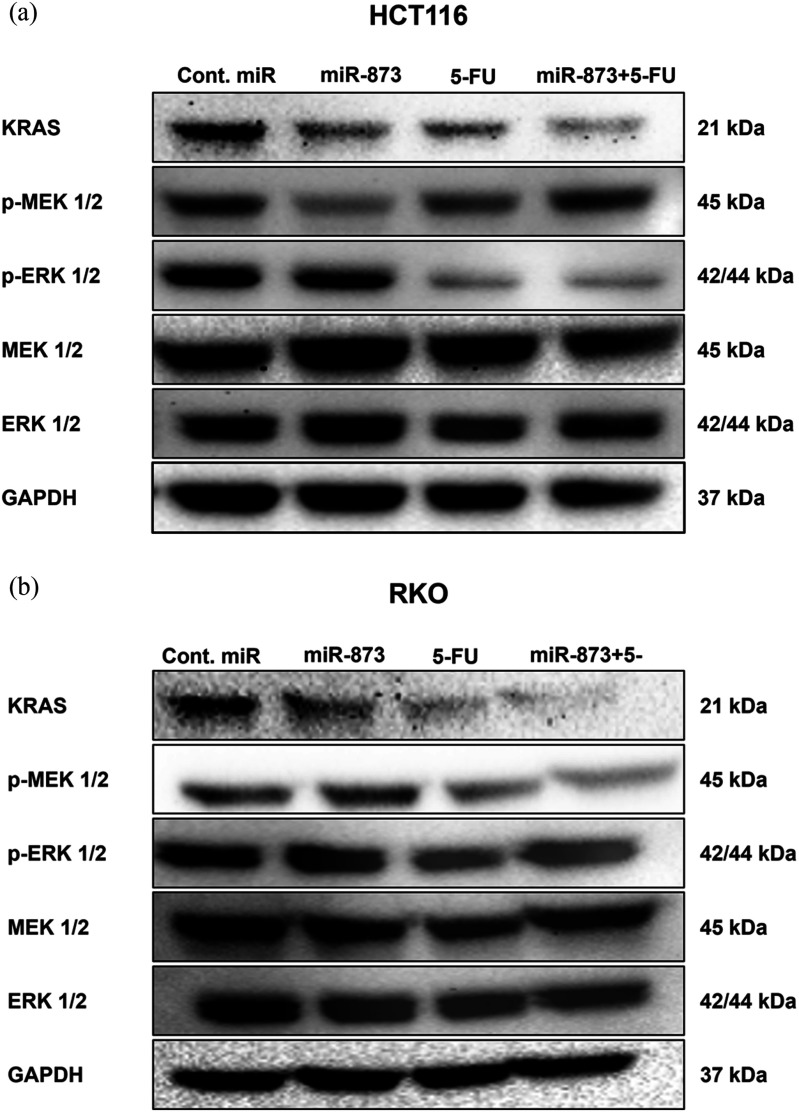
Combination treatment dampens KRAS/MAPK signaling. Immunoblots of KRAS pathway proteins after control miR, miR-873 (25 nM), 5-FU (12.5 M), or miR-873 + 5-FU. (a) HCT116 and (b) RKO show reduced KRAS and decreased phosphorylation of MEK1/2 and ERK1/2 with treatments, most notably under the combination; total MEK/ERK remains largely unchanged. GAPDH, loading control.

To explore the molecular basis of the enhanced antitumor effect of miR-873 alone and in combination with 5-FU, protein expression levels of KRAS and MAPK pathway members were assessed in HCT116 and RKO colorectal cancer cells. Western blot analysis quantified p-MEK/p-ERK and total MEK (MEK) and ERK (ERK) with normalization to GAPDH (Fig. 7(a), (b)). In HCT116, KRAS was reduced after miR-873 transfection and was further decreased upon addition of 5-FU. p-ERK was substantially reduced across treatment groups, whereas total ERK was largely unchanged, indicating reduced pathway activity via decreased phosphorylation rather than loss of total protein. p-MEK decreased sharply after miR-873 alone but remained closer to control levels with 5-FU and the combination, while total MEK varied without a consistent trend. Such divergence between p-MEK and p-ERK may reflect feedback regulation within the MAPK pathway, adaptive or compensatory signaling, and differences in phosphorylation kinetics or stability at the collection timepoint [45], [46], [47]. According to McFall et al., p-ERK appears to provide the most consistent downstream readout of MAPK signaling output under these treatment conditions [48]. A similar trend was observed in RKO cells. KRAS expression was downregulated by miR-873 and was lowest when the two treatments were combined. While total MEK and ERK remained stable, p-MEK was significantly decreased in treated samples, while p-ERK was only slightly inhibited. The findings suggest that miR-873 suppresses KRAS-dependent signaling even in wild-type KRAS cells and that its combination with 5-FU reinforces this repression. Combined, our findings show that miR-873 represses KRAS expression and reduces MEK and ERK phosphorylation in both colorectal cancer models. The addition of 5-FU further enhanced these effects, consistent with the reduced spheroid growth and cell viability observed in earlier assays.

### G. miR-873 Combined With 5-FU Engages Apoptotic and Cell-Cycle Regulatory Programs in Colorectal Spheroids

**FIGURE 8.**
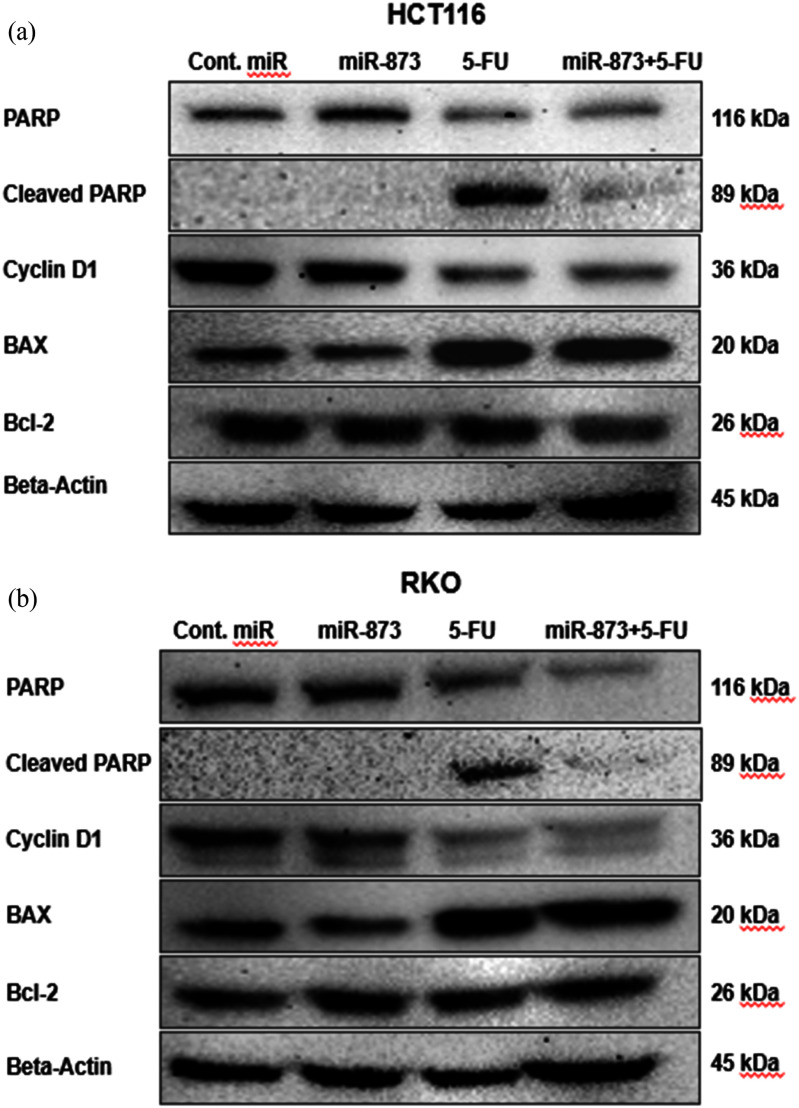
miR-873 combined with 5-FU promotes apoptosis and reduces Cyclin D1 expression in CRC spheroids. Immunoblots in (a) HCT116 and (b) RKO spheroids for PARP/cleaved PARP, Cyclin D1, Bax, and Bcl-2, with -actin as the loading control. Combination treatment increased cleaved PARP and shifted the Bax/Bcl-2 balance toward a more pro-apoptotic state while reducing Cyclin D1 expression, consistent with enhanced apoptotic signaling and suppression of proliferative capacity.

We evaluated apoptotic and cell-cyclerelated protein markers in HCT116 and RKO spheroids after treatment with miR-873, 5-FU alone or their combination (Fig. 8(a), (b)). The panel included cleaved PARP as a marker of caspase-dependent apoptosis, the Bax/Bcl-2 ratio as an indicator of mitochondrial apoptotic commitment, and Cyclin D1 as a key regulator of G1/S cell-cycle progression linked to MAPK signaling. In HCT116 spheroids, cleaved PARP increased with 5-FU and remained detectable in the combination treatment, consistent with activation of apoptotic signaling. The Bax/Bcl-2 ratio shifted toward Bax, most notably in the combination group, indicating enhanced intrinsic apoptotic priming. Cyclin D1 expression decreased with treatment and remained low under co-treatment, consistent with reduced proliferative signaling. In RKO spheroids, cleaved PARP was most strongly induced by 5-FU and remained detectable in the combination group. The Bax/Bcl-2 ratio increased following drug exposure and was highest in the dual-treatment condition. Cyclin D1 declined with 5-FU and reached its lowest level in the combination group. Taken together, both spheroid models demonstrated the strongest pro-apoptotic and anti-proliferative profile under combined miR-873 and 5-FU treatment, as evidenced by increased cleaved PARP, a shift toward a pro-apoptotic Bax/Bcl-2 balance, and reduced Cyclin D1 expression. These findings are consistent with the reduced spheroid growth and viability observed in earlier assays and further support the enhanced antitumor effect of the combination treatment.

## IV. Discussion

Advanced colorectal cancer represents a major clinical challenge. Contemporary multimodal care incorporates surgery, fluoropyrimidine-based chemotherapy, targeted agents, and immunotherapy. However, durable control in metastatic disease is infrequent, and five-year survival for stage IV remains at approximately 15 of patients [5], [7]. These poor outcomes are closely linked with intrinsic and acquired drug resistance, where pathway cross-talk and intratumoral heterogeneity undercut single-node inhibitors and limit the durability of targeted approaches [11], [12], [13], [14], [49].

In this context, our key quantitative findings include: (i) high KRAS expression predicting worse overall survival in a large CRC cohort (HR 1.27; p 0.018); (ii) robust functional suppression of clonogenicity and invasion upon KRAS inhibition and miR-873 restoration; and (iii) strong low-dose combination activity in 3D PEGDA microwells, where 25 nM miR-873 + 12.5 M 5-FU reduced spheroid expansion to 0.60.7-fold versus 2.1-fold in controls and decreased live cells to 41 in both models.

Within this landscape, KRAS is at the heart of RAFMEKERK (MAPK) signaling, which drives proliferation, invasion, and therapy resistance in CRC [11], [12], [13], [14], [17], [18]. Oncogenic activation maintains MAPK output, confers resistance to anti-EGFR antibodies, and diminishes responses to fluoropyrimidine-based chemotherapy. Higher levels of KRAS expression or mutation status are along with poorer survival and lower benefit from systemic therapy [7], [13], [17], [18], [50]. These observations support approaches that dampen KRAS signaling to surmount drug resistance, improve the effectiveness of 5-FUbased treatments, and patient survival.

MicroRNAs (miRNAs) are 22-nt regulators that silence multiple target mRNAs, letting them tune entire signaling networks. In cancer, loss of tumor-suppressive miRNAs or gain of oncomiRs drives oncogenic signaling pathways, cell-cycle, and apoptosis-evasion programs. Therefore, restoring or inhibiting specific miRNAs can rebalance multiple oncogenic pathways at once and reduce resistance driven by pathway crosstalk. As small, engineerable oligonucleotides (mimics/antimiRs) that are compatible with modern delivery platforms and combination chemotherapy, miRNAs are well-suited for therapeutic targeting and chemosensitization [51], [52].

3D platforms provide a physiologically relevant context to study such network-level control of tumorigenesis [33]. For this reason, we used a 3D platform that preserves key tumor constraints with a PEGDA-based microwell spheroid system that gives us a controllable, high-throughput 3D setting that captures diffusion gradients and matrix-imposed constraints missing in 2D, while letting us fix initial spheroid size and density for clean pharmacologic readouts with its bioinert, cell-repellent, and optically clear features. Previously, we used PEG/PEGDA microwells to fabricate arrays by UV photopolymerization and run true drug-combination tests directly on 3D Glioblastoma spheroidsdemonstrating that the platform is inexpensive, scalable, and well-suited for doseresponse and combo screening compared with hanging-drop, ULA plates, or PDMS wells [53], [54].

In this study, we investigated the role of miR-873 in targeting CRC tumorigenesis by suppressing KRAS signaling using both 2D functional assays and 3D tumor spheroid models. To our knowledge, this is the first application of our PEGDA microwell system to generate CRC tumor spheroids and a combination of miR-873 with 5-FU testing.

Our analysis in TNMplot showed that KRAS expression increases from normal to primary and metastatic colon cancer tissues, and our KaplanMeier survival analysis in 1061 patients indicated that higher KRAS associates with worse overall survival, highlighting KRAS as a clinically relevant oncogenic driver in CRC.

In order to understand the functional role of KRAS in CRC tumorigenesis, we targeted KRAS by siRNA in HCT116 (KRAS^G13D) and RKO (KRAS^WT) cells and observed suppressed viability, colony formation, and invasion. These results are consistent with prior studies, where p5RHH nanoparticledelivered KRAS siRNA or cetuximab-coupled (e)siRNAKRAS silencing diminished CRC proliferation, colony formation, and invasion [55], [56].

To target KRAS in CRC, we conducted an extensive analysis using TargetScan, a miRNA-binding prediction tool, and identified a miR-873 binding site in the KRAS mRNA 3 UTR. We demonstrated that restoring miR-873 reduced KRAS expression and impaired colony formation and invasion in both HCT116 and RKO. Prior reports linked elevated levels of miR-873 to suppression of cell viability and metastasis ability through regulation of KRAS signaling in triple-negative breast and pancreatic cancer tumor models [57]. To our knowledge, this is the first report linking miR-873 to predicted KRAS targeting and KRAS/MAPK modulation in CRC. Upon KRAS siRNA knockdown or miR-873 restoration, KRAS expression decreased, and both colony formation and invasion were suppressed in HCT116 and RKO cells, indicating that miR-873 regulates cell viability and metastatic traits through KRAS signaling. The observed larger effect after KRAS siRNA and miR-873 treatments in HCT116 is consistent with oncogenic KRAS reliance, whereas the significant response in RKO indicates genotype-independence benefit via convergent MAPK/cell-cycle and apoptotic programs [12], [58]. The stronger response in HCT116 may indicate greater reliance on oncogenic KRAS signaling.

Fluoropyrimidine responses are shaped by MAPK tone and apoptotic priming; therefore, we tested the combination of miR-873 and chemotherapeutic agent 5-FU (a backbone in FOLFOX/FOLFIRI). Prior CRC models have shown that suppressing MAPK signaling enhances the efficacy of 5-FU and oxaliplatin, supporting our objective to pair MAPK targeting strategies, such as combining miRNA restoration with fluoropyrimidines [59], [60].

To evaluate the combined effect of miR-873 and 5-FU, we conducted cell viability based on a two-drug synergy assay, and we analyzed a two-drug interaction matrix with SynergyFinder+. Our synergistic score evaluation of combinatory treatment showed highly positive synergy scores across ZIP, HSA, Bliss, and Loewe, designating a combination of miR-873 and 5-FU, even at low concentrations such as 25 nM miR-873 + 12.5 M 5-FU, in both CRC cell lines.

To investigate the effect of the best combinatory score of miR-873 and 5-FU on spheroid expansion, viability, and the underlying mechanism, we conducted a 3D tumor formation assay in our PEGDA microwell system using 25 nM miR-873 and 12.5 M 5-FU. The miR-873 + 5-FU combination produced the strongest constraints on spheroid expansion and viable counts relative to single agents: in HCT116, area fell to 0.61 0.18-fold (control 2.08 0.49; miR-873 1.16 0.18; 5-FU 1.01 0.12) and live to 40.9 1.3 (5-FU 45.8; control 100); in RKO, area reached 0.66 0.04-fold with 41.9 2.1 live cells. Morphologically, combination-treated spheroids were smaller, less cohesive, and irregular, consistent with weakened KRAS/MAPK-driven growth/adhesion. Mechanistically, Western blot analysis showed that the combination suppresses KRAS, p-MEK, and p-ERK; lowers Cyclin D1 (consistent with ERK-dependent G1/S control); and elevates apoptotic readouts, including cleaved PARP and a Bax-skewed Bax/Bcl-2 ratio, supporting a model in which ERK-driven G1/S promotion is blunted, and 5-FUinduced nucleotide/genotoxic stress more readily triggers caspase-dependent apoptosisaligning with reports that MEK/ERK modulation potentiates 5-FU/oxaliplatin in CRC [59], [60]. Moreover, these CRC findings are consistent with our earlier PEGDA-based glioblastoma work, where restoring microRNA enhanced standard chemotherapy and broadly dampened pro-growth/pro-survival signaling [36], [61], reinforcing the broader concept of miRNA-guided chemo-sensitization in 3D systems and establishing our PEGDA platform as a practical screen for combination efficacy.

Here, we showed miR-873 suppresses KRAS/MAPK expression and enhances 5-FU efficacy across genotypes, with the largest effects in KRAS-mutant HCT116 and consistent benefit in KRAS-WT RKO. The 25 nM + 12.5 M pair provides a practical starting point for KRAS 3UTR reporter assays and KRAS rescue/add-back to tighten causality, and for delivery optimization (e.g., nanoparticles or antibodyRNA conjugates) guided by RNA-therapeutic precedents. Future studies should focus on testing miR-873 + 5-FU in patient-derived organoids to see how inter-tumoral heterogeneity affects response, and to optimize delivery of the miRNA. Safety and efficacy should then be assessed in vivo using flank and orthotopic models. Work in patient-derived xenografts (PDX) can further identify predictive biomarkers of response and potential resistance mechanisms.

## V. Conclusion

Advanced CRC outcomes remain poor and underscore the need for pathway-guided chemosensitization. We show that restoring miR-873 suppresses colony formation and invasion in CRC models in a manner consistent with KRAS modulation. Combining miR-873 with 5-FU enhances anti-tumor activity synergistically and suppresses 3D PEGDA spheroid growth in both KRAS-mutant (HCT116) and KRAS-wild-type (RKO) models, accompanied by decreased KRAS/MAPK signaling output and reduced Cyclin D1. Moreover, suppression of cell viability is accompanied by increased apoptotic readouts, including cleaved PARP and a Bax-skewed Bax/Bcl-2 ratio. The low-dose combination of 25 nM miR-873 plus 12.5 M 5-FU yielded reproducible synergy and the largest decrease in viability. While our work is limited to CRC cell lines and in vitro/3D platforms, these findings suggest that miR-873 restoration, alone or in combination with 5-FU, is a KRAS/MAPK-modulating strategy that may enhance fluoropyrimidine therapy in CRC. Further testing in patient-derived and in vivo models may clarify patient selection, delivery, safety, and resistance mechanisms.
